# Ocular Involvement and Blindness Secondary to Linear IgA Dermatosis

**DOI:** 10.1155/2010/280396

**Published:** 2010-12-22

**Authors:** Cinthya Ramos-Castellón, Gabriela Ortiz-Nieva, Fernando Fresán, Leonardo Villalvazo, Yonathan Garfias, Alejandro Navas, María C. Jiménez-Martínez

**Affiliations:** ^1^Department of Cornea and Refractive Surgery, Institute of Ophthalmology “Conde de Valenciana”, 06800 Mexico City, DF, Mexico; ^2^Department of Dermatology, Institute of Ophthalmology “Conde de Valenciana”, 06800 Mexico City, DF, Mexico; ^3^Department of Pathology, Institute of Ophthalmology “Conde de Valenciana”, 06800 Mexico City, DF, Mexico; ^4^Department of Immunology and Research Unit, Institute of Ophthalmology “Conde de Valenciana”, 06800 Mexico City, DF, Mexico; ^5^Department of Biochemistry, Faculty of Medicine, UNAM, 04510 Mexico City, DF, Mexico

## Abstract

A 43-year-old man with linear immunoglobulin A (IgA) dermatosis associated with gluten intolerance presented with progressive vision loss, pain and photosensitivity in both eyes. His visual acuity was light perception (LP) in both eyes. A physical examination revealed bullous, papular lesions with erythematous borders in periocular tissues, limbs, and thorax. Slit-lamp examination showed conjunctival hyperemia, fibrosis, corneal opacification, and vascularization with epithelial defects. Immunofluorescent skin and corneal surface biopsy studies showed linear IgA deposits. The patient was treated with keratolimbal allogenic transplantation and cryopreserved amniotic membrane in the right eye. Regardless of the treatment he persisted with torpid evolution developing retinal and choroidal detachments. After these events he was started on intravenous immune globulin (IVIG) and showed very slight improvement in ocular surface. These types of blistering diseases are rare in the eye. Even when adequate local treatment is given, systemic treatment is mandatory and ocular prognosis can be unsatisfactory.

## 1. Introduction

Linear IgA dermatosis (LAD) is an autoimmune epidermal disorder that may affect skin and mucous membranes [1]. Dermatosis can be recurrent and produce purulent lesions associated with bullae or vesicles [2]. This is a rare bullous disease with ophthalmologic manifestations characterized by dry eye, foreign body sensation, conjunctival scarring with trichiasis, entropion, corneal opacification, neovascularization, and potential blindness [3]. This pathology can be difficult to distinguish from other scarring diseases such as ocular cicatricial pemphigoid [4]. To confirm the diagnosis, a skin biopsy must show deposits of IgA in a linear distribution over the junction between dermis and epidermis [2].

We describe a case of chronic cicatrizing conjunctivitis associated with gluten intolerance and dermatological involvement. Skin biopsy specimens underwent immunofluorescence, confirming LAD.

## 2. Case Report

**Figure 1:**
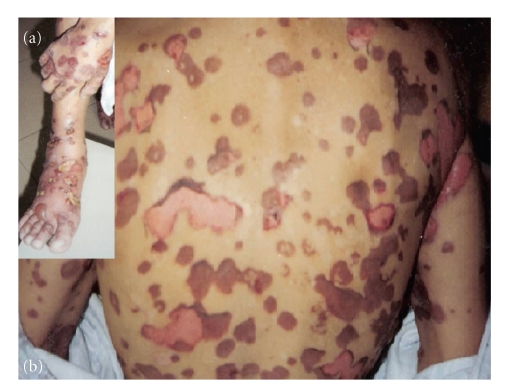
Patient with multiple dermatologic lesions distributed in limbs (a) and back (b).

A 43-year-old man with a 5-year history of gluten intolerance and bullous dermatosis had been treated for recurrent conjunctivitis. He came to us with progressive vision loss, photosensitivity, foreign body sensation, and burning sensation in both eyes. He presented bullous and papular lesions with erythematous base in periocular tissues, limbs, thorax (Figure 1). Two years before, a previous diagnosis of dermatitis herpetiformis in another center was given and was treated with 50 mg per day of oral dapsone and 5 mg per day of oral prednisolone. 

**Figure 2:**
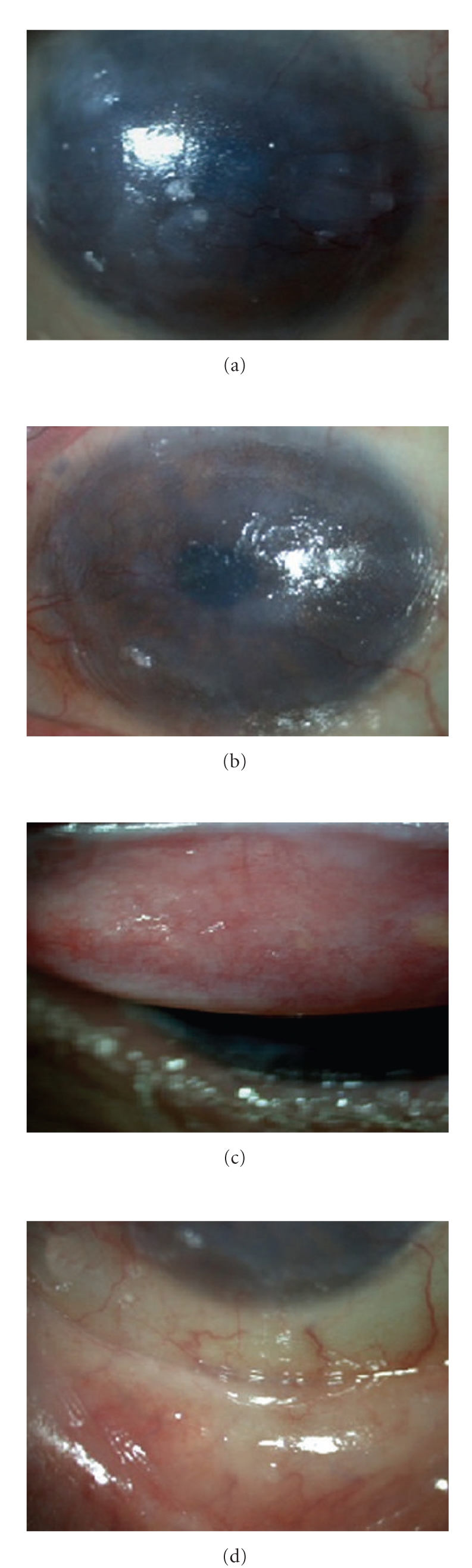
Right eye (a) and left eye (c) showing subconjunctival fibrosis, corneal opacities and vascularization as well epithelial defects. Right eye upper tarsal conjunctiva with fibrosis (b) and left eye (d) lower tarsal conjunctiva with subepithelial fibrosis.

**Figure 3:**
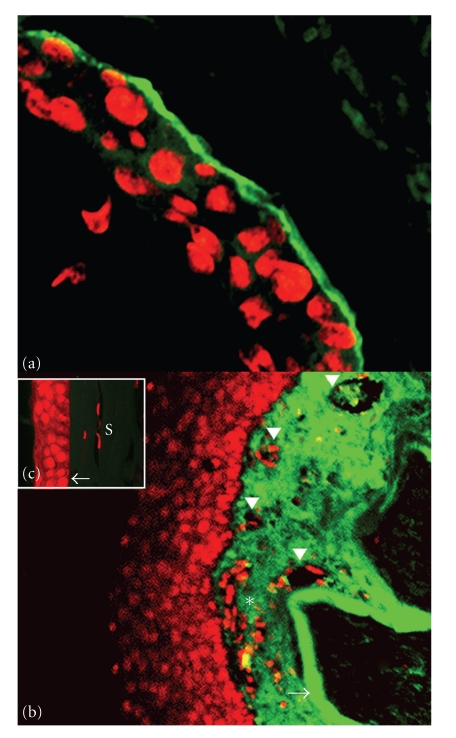
Immunofluorescence showing skin (A). Affected cornea (B) showing IgA deposits along the basal membrane and Bowman's layer (arrow) with inflammatory infiltrates (asterisk) and blood vessels (arrowheads). Comparison with healthy cornea tissue (C) showing intact Bowman's layer (arrow) and stroma (S). (Micrograph A, 400X; B, 100X, counterstain Propidium iodide).

The visual acuity was light perception in each eye. Slit lamp examination showed dysfunctional meibomian glands, conjunctival hyperemia, subconjcuntival fibrosis, corneal superficial vascularization, and opacification with epithelial defects, and the rest of the anterior segment was difficult to evaluate (Figure 2). The ocular ultrasound was unremarkable in both eyes. A histopathological examination of the skin biopsy revealed neutrophil alignment along basal membrane and subepidermal cleavage with inflammatory cells in the superficial dermis; immunofluorescence confirmed IgA deposits along the basal membrane (Figure 3(a)).

We decided to continue with dapsone and started him on a topical erythromycin ointment, lubricants, 50 mg per day of systemic prednisolone, 50 mg per day of sulfone, and 75 mg daily of azathioprine. A keratolimbal allograft transplantation was performed, with superficial keratectomy that underwent histopathological studies. We observed a complete destruction of Bowman's layer, replaced by fibrotic tissue with perforant vessels, and immunofluorescence studies confirmed IgA deposits over this area (Figure 3(b)).

**Figure 4:**
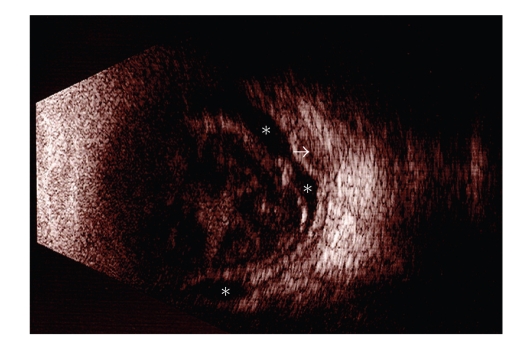
Right eye ultrasound B-scan with both retinal (asterisks) and choroidal (arrow) detachments.

**Figure 5:**
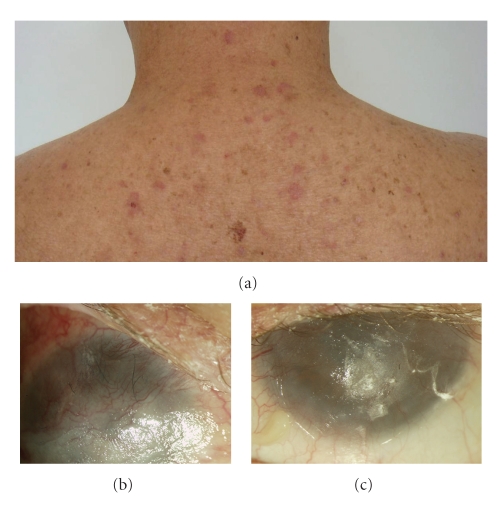
Dermatological lesions improved after treatment (a). Right eye (b) and left eye (c) ocular surface photographs after initial IVIG treatment.

A monolayer of cryopreserved amniotic membrane was implanted in his right eye. We suggested immunoglobulin therapy, but the patient could not obtain the treatment. Four months after the surgery, a corneal melt was noted and eventually he needed two more amniotic membrane patches for immanent perforation. Unfortunately he persisted with torpid evolution and the right eye visual acuity displayed LP without color discrimination and the ultrasound showed retinal and choroidal detachments (Figure 4). The left eye was not treated surgically and the visual acuity slightly improved to hand motion. Although dermatologic lesions improved significantly after IVIG therapy (Figure 5(a)), unyielding, severe ocular surface disease persisted (Figures 5(b) and 5(c)).

## 3. Discussion

We presented a case of IgA linear dermatosis with a very severe ocular and predominantly corneal affection. Although there are case reports of LAD without skin or mucous membrane involvement, the predominant dermatologic findings in this case prompted our diagnostic consideration of LAD [1, 3]. 

The dermatologic lesions resembling LAD are dermatitis herpetiformis, pemphigoid, epidermolysis bullosa acquisita, and ocular pemphigus vulgaris. The differences between these diseases was defined almost thirty years ago [2, 5, 6]. The bullous diseases develop ocular manifestations usually resistant to surgical and medical treatments. In this case, despite good compliance and multidisciplinary approach, the ocular outcome was catastrophic [4].

The average incidence is around 0.58 cases per 200,000 individuals. Previous reports indicate severe ocular affection and progression in men, thus the presence of ocular disease can be more aggressive in the masculine gender such as in this case [6]. Nevertheless LAD also affects the female and pediatric population [7].

In this case there were no previous medications or vaccinations associated with drug-induced linear IgA dermatosis [8, 9].

Despite that, theoretically, end-stage disease is rare in LAD [9], and even though we treated the patient with systemic immunosuppression agents and surgical procedures the ocular response was very poor. There are previous reports of corneal perforations [1, 4] and the use of corneal transplantation and limbal stem cell grafts [4], but in those cases the outcomes were also unsatisfactory.

To our knowledge, there are no previous reports about the use of amniotic membrane in this disease, but after the outcome of our patient, it is hard to know how useful or dangerous it can be in these kinds of cases.

Based on evidence that the basement membrane type IV collagen is a target for the IgA autoantibodies in other tissues [10], we performed and were able to elicit positive immunoflorescence studies on the corneal epithelium. We therefore suppose that type IV collagen of corneal epithelium, Bowman's membrane may be an IgA auto antibody target in cases of LAD.

Even though up to 50% of the patients with LAD presented ocular involvement [1, 2, 6, 9, 11], there is scanty literature in the ophthalmologic field [2, 4, 11]. 

Unlike dermatitis herpetiformis and other dermatologic conditions which are linked to gluten enteropathy, LAD is not associated with gluten intake [2]. The patient used to avoid gluten in his diet but patients with LAD tend to not respond to a diet free of gluten as in other dermatological diseases [2, 12]. 

Despite the fact that patients with LAD can respond to IVIG [3], in severe cases like this patient, it is hard to expect an evident respond. 

In conclusion linear IgA can be a devastating problem for the ocular surface. As ophthalmologists, we need to be prepared for these scenarios and be aware of the unfavorable presentations of this disease.
